# Changes in bone density and bone quality caused by single fasting for 96 hours in rats

**DOI:** 10.7717/peerj.6161

**Published:** 2019-01-09

**Authors:** Yuko Hisatomi, Kenji Kugino

**Affiliations:** Division of Nutritional Science, Graduate School of Human Health Science, University of Nagasaki, Nagasaki, Japan

**Keywords:** Fasting, Bone quality, Bone density, Contrast enhanced micro-CT, The minimum cross-sectional moment, The polar moment, Rat, Lumbar vertebrae

## Abstract

**Background:**

Young women occasionally engage in dietary restrictions accompanied by fasting for the purpose of losing weight, but such restrictions have various effects on body functions. The recent increase in the number of people suffering from osteoporosis has become a major social problem mainly in industrialized countries.Therefore, we think it is important to understand the effects of fasting on bone vulnerability, especially to bone quality.

**Methods:**

Animals used male Wister rats weighing 130 g (6 weeks of age), and were divided into a control group (*n* = 5) and a fasting group (*n* = 6). The experimental period was 14 days, the control group had ad libitum food throughout the experimental period, the fasted group was fasted for 4 days, and than, had ad libitum food for 10 days. In this study, parameters related to bone fragility due to three-dimensional bone architecture were determined on Contrast enhanced micro-CT images of the lumbar spine and were used as a method for the evaluation of bone quality. In addition, a time-course observation of each individual was carried out during the fasting period and later upon resuming food intake. Cross-sectional images of all vertebrae were obtained from radiographic computed tomography and were analyzed by using Latheta software ver. 3.0 (Hitachi-Aloka Medical, Nagasaki, Japan). The region of interest that was misrecognized in each cross-sectional image was made consistent with the anatomical structure by carrying out corrections manually and by identifying the cortical bone areas and cancellous bone areas.

**Results:**

Our findings showed that while single fasting for 96 h did not cause any major change in the macroscopic morphology of bone, it caused a marked decrease in bone density. In addition, the minimum cross-sectional moment, which indicated the “strength against bending” as well as the polar moment that indicated the “strength against torsion” were both lower than in non-fasted rats. Further, after resumption of feeding, bone mineral content in the fasting group recovered rapidly and starting at day 4 after resumption of feeding, there was no difference with the control group. On the other hand, the values of the minimum cross-sectional moment and polar moment did not recover, and the difference with the control group increased during the feeding period.

**Discussion:**

On the basis of this study, the authors estimate that the fasting-induced decrease in bone minimum cross-sectional moment and polar moment may have been due to changes affecting some factors involved in bone quality, and thus could be useful as a parameter in future studies aimed at elucidating bone quality. At least, in the case where bone change accompanied with a change in macroscopic distribution of mineral components occurs, the values of minimum cross-sectional moment and polar moment are considered to be bone parameters that will provide valuable information to elucidate bone quality.

## Introduction

Young adolescents, particularly women, occasionally engage in dietary restrictions accompanied by fasting for the purpose of losing weight, but such restrictions have various effects on body functions. In previous study by the authors and others, young rats (5–6 weeks of age) were subjected to 4 days of fasting once or twice, and observed the various effects of fasting. As a result, extreme dietary restrictions (fasting) had negative impacts on bone, muscle and digestive organ function such as organ atrophy and decreasing bone density (Hisatomi et al., 2013a; Kugino, 1995). Further on, in an experiment in which the C57BL/6J male mice (16 weeks of age) were fasted for 3 days, optical microscopic observation of mouse brain tissues has shown that fasting causes abnormal behavior in mice and suppresses neurogenesis in the hippocampal dentate gyrus and in the subventricular zone. The findings also suggested that fasting inhibits the neuronal neogenesis in the subventricular zone and hippocampus of the brain, causing damage to brain functions (Hisatomi et al., 2013b). As fasting results in changes in the musculoskeletal system and digestive system as well as in other systems, the possibility of irreversible changes in the associated histological structures needs to be considered.

The recent increase in the number of people suffering from osteoporosis has become a major social problem mainly in industrialized countries. Because of the need for preventive measures against this condition, we have been proceeding with a particular research focus on the impact of fasting on bone fragility. Several previous studies on bones have shown that after bone mass reaches its maximum in adulthood around the age of 20 years, it decreases with age; therefore, finding out how to further increase the maximum bone mass is believed to be the most important method of osteoporosis prevention (Kröger et al., 1993). Improvement of eating habits during the period of growth and development until puberty has been found to increase bone mass (Theintz et al., 1992); therefore, proper nutritional support until the age of puberty is considered extremely important to increase each individual’s maximum bone mass. Meanwhile, in young adolescents, weight-loss behaviors caused by a strong desire to have a slim body have occasionally been found in women (Sano et al., 2008). The desire to have a slim body is often found in adolescents in their early teens. Previous reports have also shown that approximately half of elementary school fifth graders and nearly all middle school students had a desire to have a slim body, and that about 20% and about 40% of such individuals had already experienced dietary restrictions aimed at losing weight (Kaneko et al., 1999). Although there are concerns that dietary restrictions associated with fasting for the purpose of slimming (weight loss) during the growth period before and throughout puberty may increase the risk of developing osteoporosis after reaching adulthood, no previous report has provided any clear evidence that this was true in humans. However, through experiments conducted on laboratory animals, the authors have found that changes in calcium balances due to fasting inhibited the growth of the femoral cortical bone, that the changes had a great impact on the decrease in bone density and weakening of bone strength after reaching maturity, and that the impact of fasting was more severe in younger individuals (Hisatomi et al., 2013a). Thus far, the authors have been carrying out a study of the effects of fasting on bones by conducting a follow-up of individuals and by using bone mass (bone density) as the main non-invasive indicator. However, in the present study, the focus was on the changes in bone quality which, in combination with bone mass, was an important factor for the determination of bone strength. In the experiments described in this study, parameters related to bone fragility due to three-dimensional bone architecture were determined on Contrast enhanced micro-CT (CECT) images of the lumbar spine and were used as a method for the evaluation of bone quality. In addition, a time-course observation of each individual was carried out during the fasting period and later upon resuming food intake.

## Methods and materials

**Figure 1:**
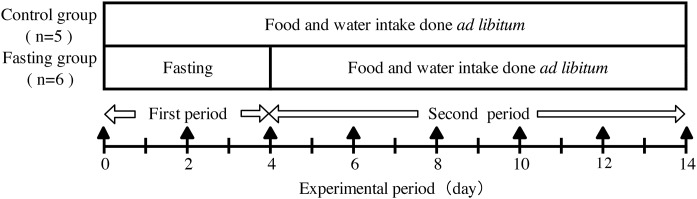
Experimental design. In the experimental period, body weight, food intake, and water intake of the rats were measured daily. Contrast enhanced micro-CT (CECT) imaging was performed every other day during the experiment period as indicated by ▴ in the figure.

The experimental design is shown in Fig. 1. Male Wister rats weighing 130 g (6 weeks of age, obtained from CLEA Japan, Nagasaki, Japan) were divided into a control group (*n* = 5) and a fasting group (*n* = 6). During the experimental period, rats had ad libitum access to experimental diet (MF-diet made by Oriental-yeast, Nagasaki, Japan) and water. During fasting also water intake was free. The compositions of the experimental diet are shown in Table 1. All animals keep in separate cages during the experiment. This study was approved by the Animal Use Committee of University of Nagasaki (approval number, 24-17) and was carried out as per the guidelines for animal experiments of University of Nagasaki and Law No. 105 and Notification No. 6 of the Government of Japan.

**Table 1:** Composition of experimental diets (g/100 g).

Ingredient	Amount
Moisture	7.7
Crude protein	23.6
Crude fat	5.3
Crude fiber	2.9
Crude ash	6.1
Nitrogen free extract	51.0
Total energy (Kcal/100 g)	360

**Figure 2:**
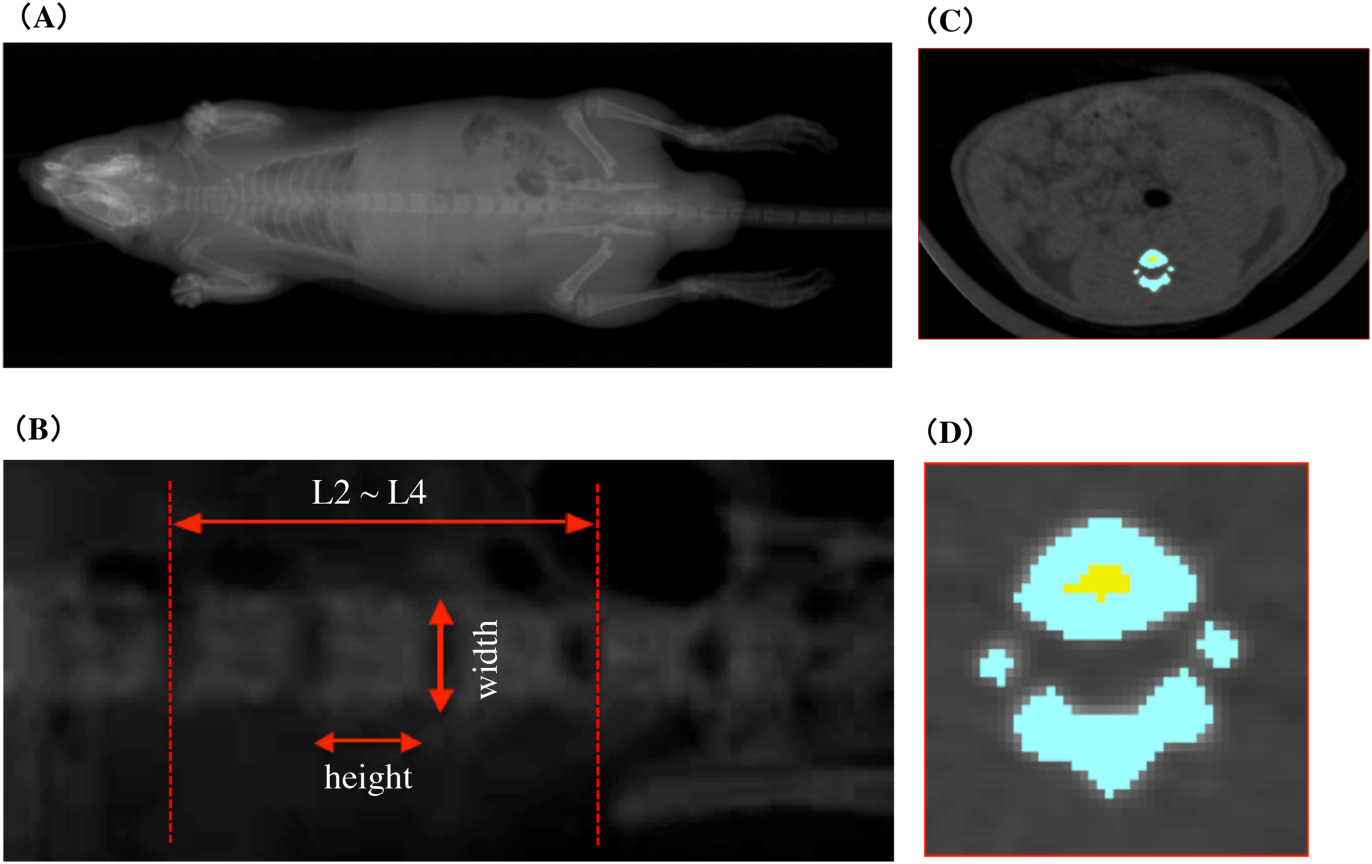
CECT scout image and measurement area. (A) The pre-scanned scout image of the rat whole body. (B) Second, third and fourth lumbar scout images of lumbar vertebral body height and vertebral body width measurements. (C) Tomographic image of lumbar vertebrae subjected to image analysis processing. The color area indicates the vertebra. (D) An enlarged image of a tomographic image of the lumbar vertebrae in (C). Blue area shows cortical bone and yellow area shows cancellous bone.

In vivo CECT imaging was performed with an X-ray CT system (Latheta LCT-100; Hitachi Aloka Medical, Nagasaki, Japan) developed for imaging small experimental animals. To perform X-ray CT scans, rats were anesthetized with isoflurane using anesthesia machine for animals (SN-487; Shinano Mfg, Nagasaki, Japan). Isoflurane anesthesia was maintained even during 30-min imaging in the X-ray irradiation box of the X-ray CT system. Imaging was performed under the setting of X-ray tube type HR-50PC, tube voltage 35.50 kV, sensible current one mA. The rats were placed in a supine position and the trunk from the diaphragm to the neck of the femur was sectioned at one mm intervals. CECT scout image and measurement area are shown in the Fig. 2. Vertebral body height and vertebral body width of lumbar vertebral body were measured from X-ray scout images. The measurement site of the vertebral body height and width was determined with reference to the illustration demonstrating the measurement of the vertebrae of Shi et al.’s (1999) Figure 1. The vertebral body height was adopted an intermediate value between superior sagittal diameter and inferior sagittal diameter. The vertebral body width was adopted an intermediate value between superior transverse diameter and inferior transverse diameter. In addition, average values of second lumbar vertebra (L2), third lumbar vertebra (L3), and fourth lumbar vertebra (L4) were adopted for the vertebral body height and the vertebral body width of each individual. Cross-sectional imaging data obtained from CECT was analyzed and processed by using LaTheta software ver. 3.0 (Hitachi Aloka Medical). Figures 2C and 2D shows cross-sectional tomographic images from rats that were subjected to image analysis and processing. The area colored in blue shows the cortical bone, and the area colored in yellow shows the cancellous bone. Cross-sectional images of all vertebrae were obtained from radiographic computed tomography and were subjected to automatic recognition by software. The region of interest that was misrecognized in each cross-sectional image was made consistent with the anatomical structure by carrying out corrections manually and by identifying the cortical bone areas and cancellous bone areas. Approximately 18 cross-sectional tomographic images from the second lumbar vertebra to the fourth lumbar vertebrae of rats were subjected to image analysis. As described above, each bone site was specified, and the mean cortical bone thickness was calculated; in addition, the following bone parameters were also calculated. The overall vertebral bone mineral density (BMD) was considered as the bone mineral content per cubic volume (mg/cm^3^) based on the following equation. The cubic volume was calculated as the integrated value of “the planar dimensions of the area of the cross-sectional tomographic image (*S*(*i*))” and “the inter-slice intervals between the cross-sectional tomographic images (*d*)”.

$${\rm Density\ }\left( {{\rm mg}/{\rm c}{{\rm m}^3}} \right){\rm } = {\rm Bone\ mineral\ content\ }\left( {{\rm mg}} \right){\rm } \div {\rm Bone\ volume\ }\left( {{\rm c}{{\rm m}^3}} \right)$$

$${\rm Bone\ volume\ }\left( {{\rm c}{{\rm m}^3}} \right){\rm } = {\rm }\Sigma S\left( i \right)\times {\rm }d{\rm } = {\rm }d\Sigma S\left( i \right)$$

The minimum cross-sectional moment of inertia (mg · cm), which was an indicator of mechanical strength in terms of flexural strength, was calculated according to the following equation. The higher its value was, the stronger the mechanical strength against flexure (Van Der Meulen, Jepsen & Mikic, 2001).

(1) $${\rm{Minimum\ cross-sectional\ moment}}\ ({\rm{mg}} \cdot {\rm{cm}}) = \int\!\!\!\int {r{\prime} \times r{\prime} \times {\rm{BMD\ d}}x{\rm{d}}y}$$

*r*′: the distance from a straight line passing through the center of gravity (*X_g_*, *Y_g_*) with respect to the image elements of a bone (*x_i_*, *y_j_*) on a radiographic image. This value depends on the angle θ of the straight line passing through the center of gravity, and it can be determined on the basis of the following equation.

$$r^{\prime} {\rm } = {\rm }R{\rm \theta } = \displaystyle{{\left| \right.{{\left({{\rm tan\theta } } \right)}^2}\; \times \; \,{x_i}\; - \,{y_j}{\rm \; } - {\rm \; tan\theta }\,\; \times \; {X_g}\; + \,{Y_g}\left. \right|} \over {\sqrt {{{\left( {{\rm tan\theta }} \right)}^2}{\rm \; } + \,1\; } \; }}\,\left( {{\rm cm}} \right)$$

BMD: Bone mineral density of a bone region (*x_i_*, *y_j_*).

The polar moment of inertia (mg · cm), which is the mechanical strength of the torsional strength, can be calculated based on the following equation, and a higher polar moment of inertia indicates stronger action against torsion (Van Der Meulen, Jepsen & Mikic, 2001).

(2) $${\rm Polar\ moment\ }({\rm mg}\,\cdot \,{\rm cm}){\rm } = {\rm \int\!\!\!\int }r{\rm } \times {\rm }r{\rm } \times {\rm BMD\ d}x{\rm d}y$$

*r*: distance from the center of gravity (*X_g_*, *Y_g_*) to the image element of a bone (*x_i_*, *y_j_*) on a radiographic image. It can be determined on the basis of the following equation.

$$r{\rm } = \sqrt {{{( {{x_i} - {X_g}} )}^2} + {{({y_i} - {Y_g})}^2}}$$

BMD: Bone mineral density of a bone region (*x_i_*, *y_j_*).

All data are presented as means ± SD. The statistical analyses were performed using excel analysis tool (Microsoft^®^ Excel for Mac Version 16.15). Student’s *t*-test was used to compare two groups. Difference was assessed with two-side test with an alpha level of 0.05.

## Results

**Figure 3:**
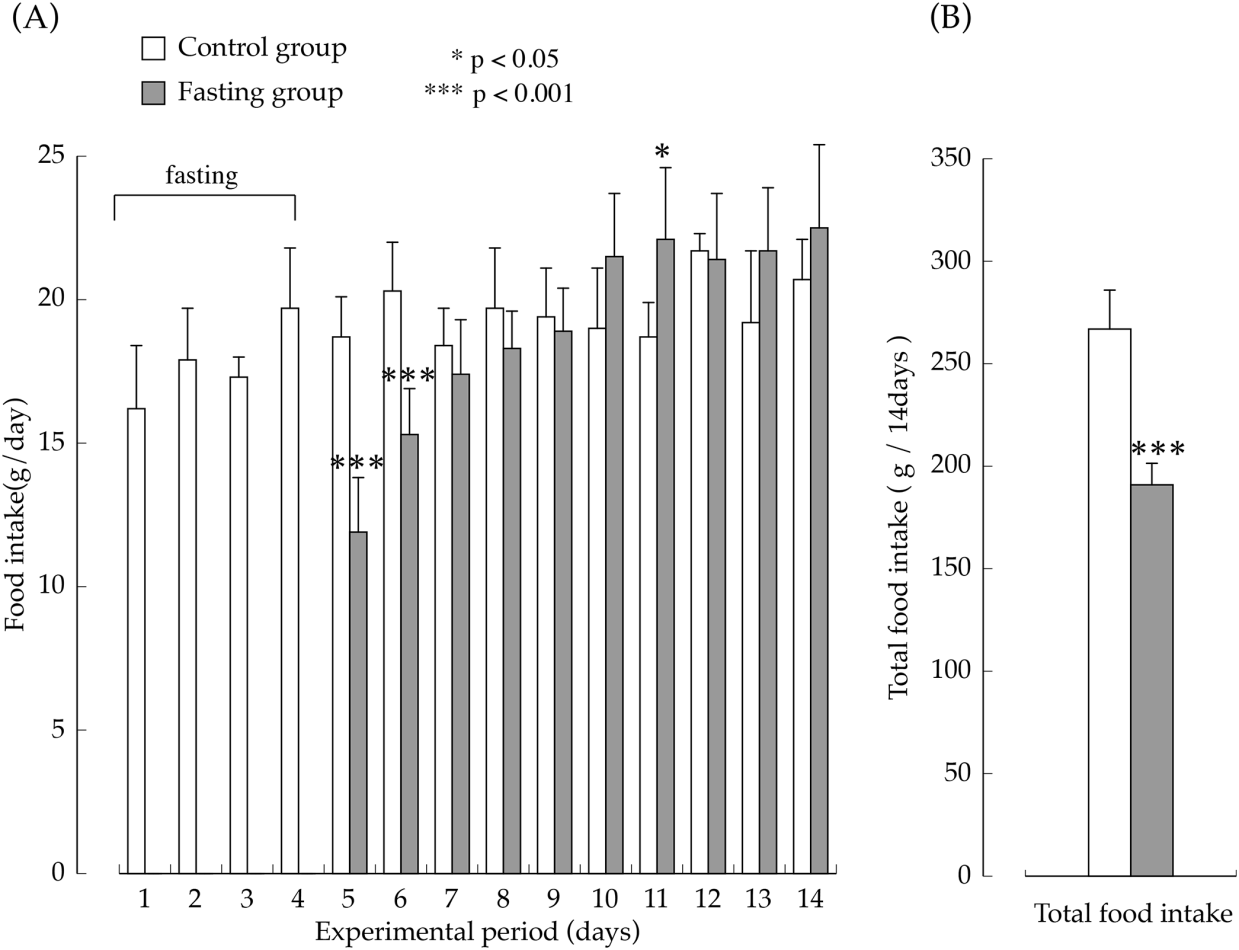
Food intake of rats in the experimental period. (A) Changes of the rats food consumption during the experimental period. (B) The total amount of food intake for 14 days. Values are Means ± SD of control group (*n* = 5) and fasting group (*n* = 6). Student’s *t*-test was used to compare two groups. *p < 0.05, ***p < 0.001, significantly different from the control value.

The amount of food intake in the two groups during the feeding period are shown in Fig. 3. The amount of food intake by the fasting group on the first day after resumption of feeding was the equivalent of approximately 60% of the amount of food intake in the control group, but later, it increased rapidly, and on the seventh day after resumption of feeding, food consumption was significantly higher than that found in the control group. The total amount of food intake in the fasting group during the entire feeding period was the equivalent of approximately 70% that of the control group.

**Figure 4:**
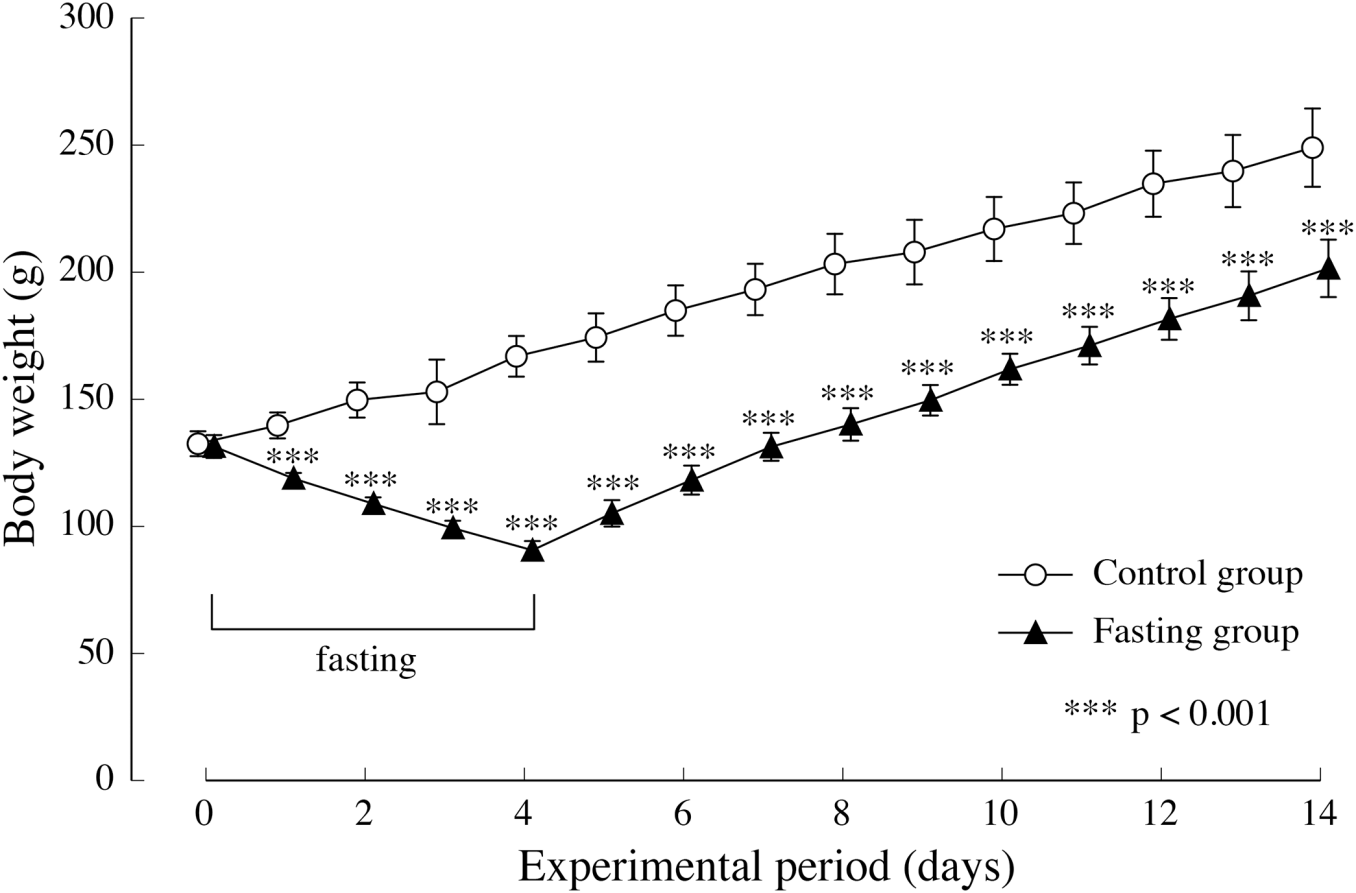
Body weight of rats in the experimental period. Values are means ± SD of control group (*n* = 5) and fasting group (*n* = 6). Student’s *t*-test was used to compare two groups. ***p < 0.001, significantly different from the control value.

Figure 4 shows the changes in body weight in the two groups. After a 4-day fasting period, body weight in the fasting group decreased from approximately 130 g to approximately 90 g, namely 55% of the body weight found in the control group. In the fasting group, weight gain after resumption of feeding surpassed that of the control group, and although the difference between the two groups decreased gradually, significant differences were also found at the end of the feeding period.

**Figure 5:**
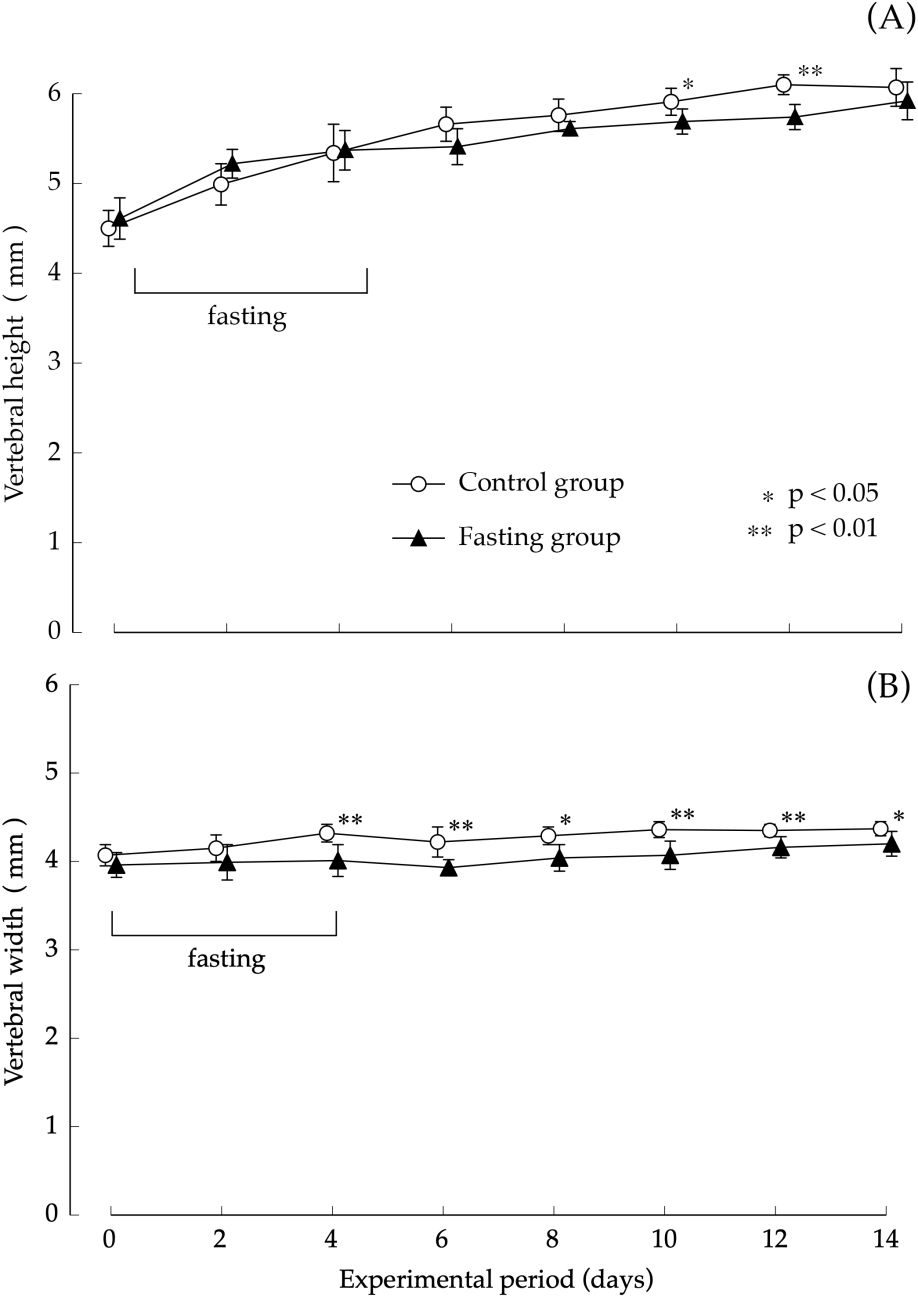
Vertebral body height and vertebral body width of rats in the experimental period. (A) Changes of the vertebral body height of rats during the experiment period. (B) Changes of the vertebral body width of rats during the experiment period. Values are means ± SD of control group (*n* = 5) and fasting group (*n* = 6). Student’s *t*-test was used to compare two groups. *p < 0.05, **p < 0.01, significantly different from the control value.

Findings pertaining to vertebral body height and vertebral body width are shown in Fig. 5. In the control group, the lumbar vertebral body height increased gradually during the study period. Although elongation was found in the fasting group after fasting, comparison with the control group showed no significant difference at the end of the feeding period. The elongation of the lumbar spine width was moderate in both groups. Values found in the fasting group after the fasting period were lower than those found in the control group, and significant differences were also found at the end of the feeding period.

**Figure 6:**
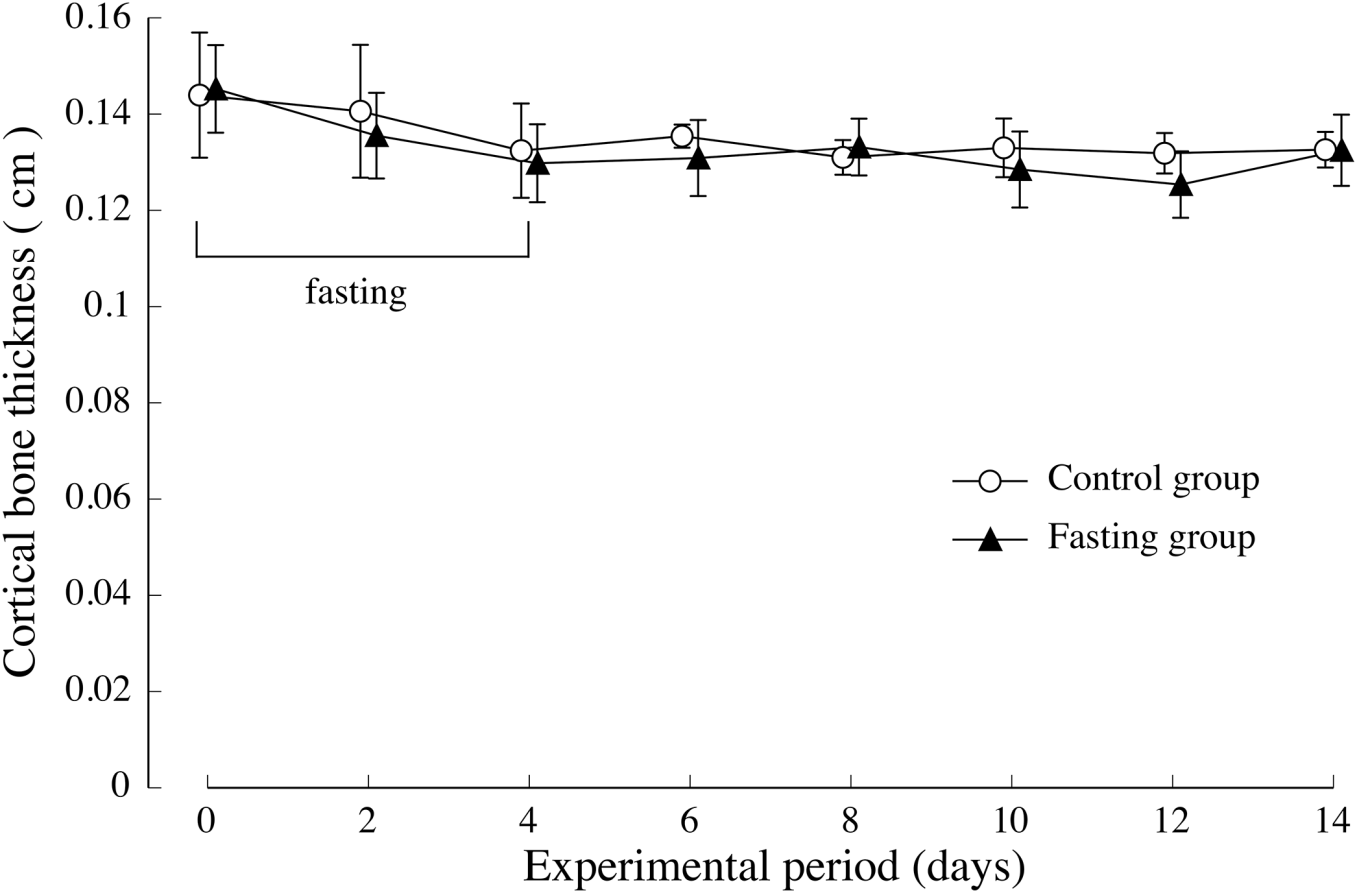
Cortical bone thickness of rats in the experimental period. The average values of second lumbar vertebra (L2), third lumbar vertebra (L3), and fourth lumbar vertebra (L4) were adopted for the cortical bone thickness of each individual. Values are means ± SD of control group (*n* = 5) and fasting group (*n* = 6). Student’s *t*-test was used to compare two groups. There was no significant difference between the two groups.

The thickness of the lumbar cortical bone is shown in Fig. 6. Regarding the cortical bone thickness, there was no significance between the control group and the fasting group, and no noticeable change was observed throughout the test period.

**Figure 7:**
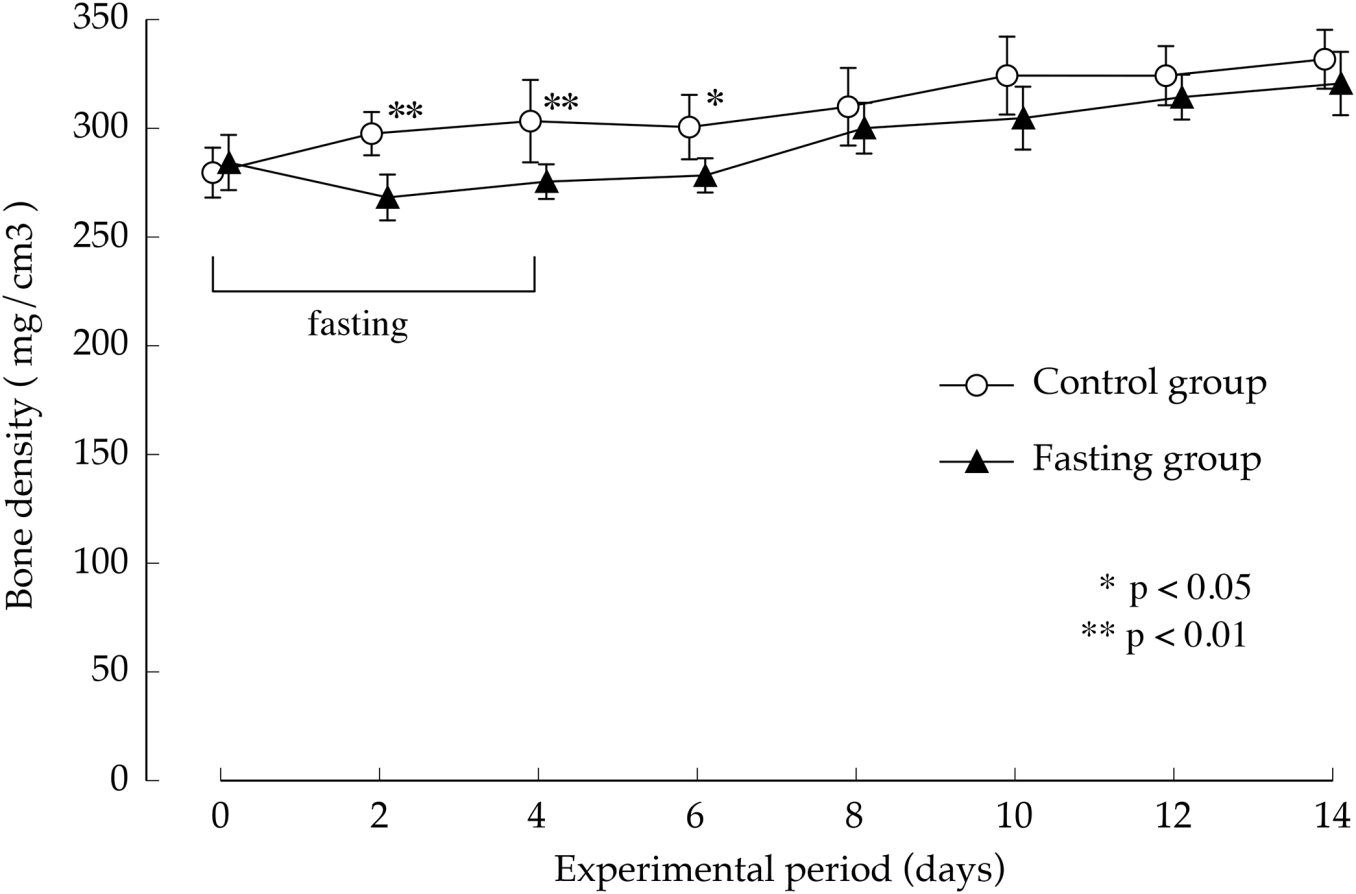
Lumbar vertebral bone mineral density of rats in the experimental period. Values are means ± SD of control group (*n* = 5) and fasting group (*n* = 6). Student’s *t*-test was used to compare two groups. *p < 0.5, **p < 0.01, significantly different from the control value.

Figure 7 shows the lumbar vertebral bone density. In the control group, bone density increased gradually during the study period. While fasting, the increase in bone density in the fasting group was inhibited and showed significantly lower values than those found in the control group, until day 2 after resumption of feeding, BMD is significantly lower in fasted group. Thereafter, BMD in the fasted group was lower than the control group, no significant difference was found between the groups.

**Figure 8:**
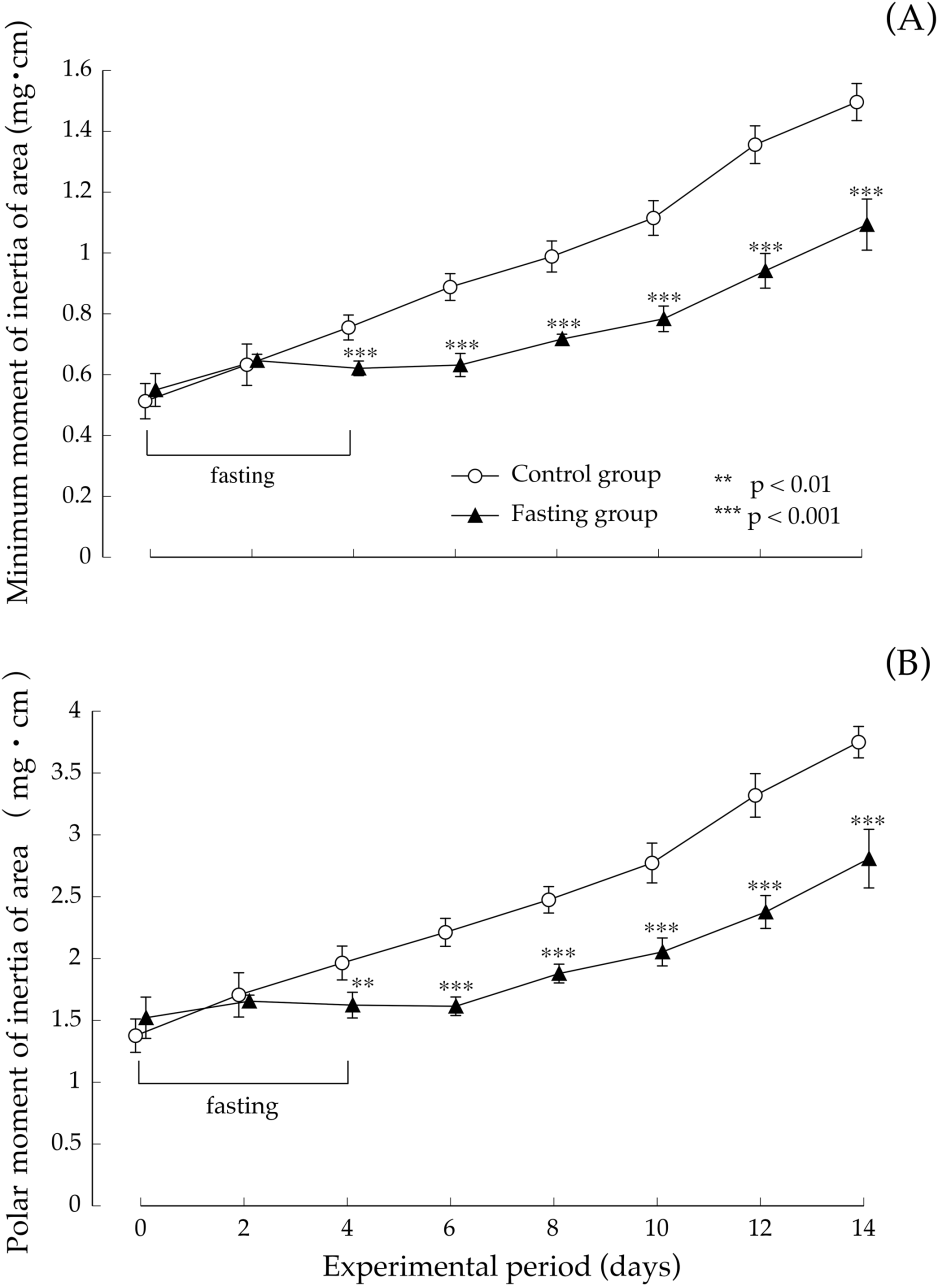
Minimum cross-sectional moment and polar moment of rats in the experimental period. (A) Changes of the minimum moment of rats during the experiment period. (B) Changes of the polar moment of rats during the experiment period. Values are means ± SD of control group (*n* = 5) and fasting group (*n* = 6). Student’s *t*-test was used to compare two groups. **p < 0.01, ***p < 0.001, significantly different from the control value.

Figure 8 shows the minimum cross-sectional moment and polar moment. In the control group, the minimum cross-sectional moment increased in a nearly linear manner during the study period. On the other hand, in the fasting group, the minimum cross-sectional moment was remarkably suppressed by fasting. After that, it began to gradually increase from the eighth day. The difference between the two groups continued to increase throughout the feeding period. Findings pertaining to the polar moment were also similar to those pertaining to the minimum cross-sectional moment. In other words, the increase in minimum cross-sectional moment and polar moment was suppressed by fasting, and even after resumption of feeding, recovery did not progress, and the difference with the control group continued to increase.

## Discussion

In this study, our experiments have shown that after 4 days of fasting, the lumbar vertebral body height and vertebral body width were slightly lower than those found in the control group; nonetheless, the difference was small, and after resumption of feeding, recovery progressed in the fasting group. At the end of the feeding period, the findings showed no difference with the control group, in the lumbar vertebral body height. But the vertebral body width was slightly significant differences in both groups. Regarding cortical bone thickness, no significant difference was found while fasting or after resumption of feeding. Meanwhile, bone density after a 4-day fasting period, which was an indicator of bone mass, was significantly lower than that found in the control group. Our findings showed that while single fasting for 96 h did not cause any major change in macroscopic morphology of the vertebrae, it caused a marked decrease in bone density. In addition, the minimum cross-sectional moment, which indicated the “strength against bending” as well as the polar moment that indicated the “strength against torsion” were both lower than in non-fasted rats. As found on the basis of the calculation method, the values of the minimum cross-sectional secondary moment and that of the cross-sectional secondary polar moment were dependent on the macroscopic distribution of mineral components, and could be one of the parameters of bone quality that reflected the bones’ macroscopic structures. The findings of our study suggested that a 4-day fasting not only caused a decrease in lumbar vertebral bone density, but also triggered changes in bone quality, and the combination of the two was estimated to be responsible for weakening the bones. Further, after resumption of feeding, bone mineral content in the fasting group recovered rapidly and starting at day 4 after resumption of feeding, there was no difference with the control group. On the other hand, the values of the minimum cross-sectional moment and polar moment did not recover, and the difference with the control group increased with the prolongation of the feeding period. In other words, our findings suggested that bone mass, which decreased due to fasting, recovered immediately after resumption of feeding, whereas bone quality, which underwent changes due to fasting, did not easily recover, even after resumption of feeding. In order to exclude the influence of the sex cycle from the experimental results, we conducted experiments using male rats in this study, but it is unlikely that there is a sex difference on the effects of fasting on bone. Therefore, the authors think that the findings obtained in this study can be extrapolated to both sexes. Bone strength consists of two factors: bone mass and bone quality. Therefore, the fact that bone quality does not easily recover suggests that bone strength does not recover. Saito, Fujii & Marumo (2006) also previously reported that bone quality played an important role in bone strength (Saito, 2010; Saito & Marumo, 2010). They indicated that bone density was significantly higher in type-II diabetes patients than in the control group. They found that the risk of vertebral body fracture was significantly higher in the diabetes group, that the degradation of bone quality was the major factor to bone fragility in type II diabetes, and that the changes in bone quality consisted of a deterioration of collagen fibers. Cross-linkages that bind collagen fibers among themselves include a cross-linking of advanced glycation end-products (“AGEs cross-linking”), such as pentosidine, which forms in the presence of elevated blood glucose levels and enzyme-dependent “physiological cross-linking”. They estimated that the deterioration of bone strength resulted from an increased “AGEs cross-linking” due to enhanced production of AGEs caused by elevated blood glucose levels. It is difficult to clearly explain the specific changes in bone architecture and describe how the low minimum cross-sectional moment and polar moment found in this study meant bone quality. In these experiments, the bone quality changes caused by fasting might be unrelated to the collagen deterioration indicated by Saito, Fujii & Marumo (2006) which are changes in bone structure at the molecular level. As shown in the calculation formula, the values of minimum cross-sectional moment and polar moment are dependent on the macroscopic distribution of mineral components; therefore, they can be understood as parameters of bone quality in terms of bone macrostructure. Therefore, a decrease in minimum cross-sectional moment and polar moment suggests a decrease in bone strength due to macromorphological changes in the bones. This suggests that the change in bone quality might be due to other causes of bone fragility, such as changes in the cross-linking structure of collagen, which are considered as more microscopic morphological changes.

The decrease in minimum cross-sectional moment and polar moment shown in this study suggests a change in the bone factor accompanied by a change in macroscopic distribution of mineral components. For example, in case of cancellous bone, it may suggest that a structural change is occurring in the trabecular bone that plays the role of beams and pillars in buildings to maintain bone strength. The cancellous bone structure must be intact to serve as supportive tissue supporting weight. However, fragmentation, irregularization, etc., might have occurred in the trabecular bone that was rapidly reconstructed during bone mass recovery after fasting. Degradation of the trabecular structure such as partial thinning and thickening of cancellous bone or deterioration of connectivity deteriorates the bone quality as a structural characteristic and causes a decrease in bone strength as a result. Also, in the case of cortical bone, bones are ossified by regularly depositing calcium phosphate on osteoid collagen fibers made by osteoblasts. However, calcium phosphate does not regularly deposit on the collagen fibers in the rapidly reconstructed cortical bone during bone mass recovery after fasting, which may impede well-balanced ossification.

Although it is difficult to determine what type of transformation of bone architecture is behind the fasting-induced changes in bone quality shown by our study, the long-term absence of recovery of the changes in bone quality (decreased bone strength) caused by a few days of fasting, despite resumption of feeding, is a noteworthy finding in terms of the nutritional aspects of the prevention of osteoporosis. The increase in the incidence of osteoporosis has recently become a major social issue in developed countries. At the same time, the finding of weight-loss behavior accompanied by fasting in many adolescent youths has also been viewed as a problem. If adolescent youths’ fasting behavior leads to changes in bone quality that is difficult to recover easily, fasting repeatedly during the period of growth may increase the risk of developing osteoporosis in the future. Previous reports have shown that in patients with amenorrhea and extreme weight loss, such as in anorexia which is often found in young women in their 20 s or younger, an age that is extremely important for bone formation, and it was reported that AN onset before 18 years of age has a lower bone mass than AN onset at a later age (Biller et al., 1989). This can occur even after increasing the amount of meal intake, after improvement of symptoms, and after recovery of body weight. For bones that were temporarily weakened and had an irregular structure due to extremely severe acute malnutrition resulting from fasting, such bones will have trouble recovering spontaneously to their original intact bone architecture, even if there is a subsequent improvement in the nutritional status.

Osteoporosis is a disease that presents with bone fragility. Thus far, it has been believed to be primarily due to a decrease in bone mass (bone density). For this reason, the current treatment and prevention options are mainly aimed at increasing bone density; however, bone strength depends not only on bone mass but also on bone quality. Currently, much remains unknown regarding the characteristics of bone architecture that is meaningful to bone quality, but in the treatment of osteoporosis by using bone resorption inhibitors, a previous report (Wasnich & Miller, 2000; Ettinger et al., 1999; Sarkar et al., 2002) has shown that the incidence of fractures decreased even in the absence of apparent increase in bone density and suggested that the so-called bone quality (which involves various factors such as bone microarchitecture, bone turnover, bone microdamage, and calcification) might play an important role in bone strength. A growing number of researchers consider that for the prevention of osteoporosis, studies aimed at elucidating bone quality will be even more important in the future (Boskey & Myers, 2004; Saito, Fujii & Marumo, 2006). On the basis of this study, the authors estimate that the fasting-induced decrease in bone minimum cross-sectional moment and polar moment may have been due to changes affecting some factors involved in bone quality, and thus could be useful as a parameter in future studies aimed at elucidating bone quality. At least, in the case where bone change accompanied with a change in macroscopic distribution of mineral components occurs, the values of minimum cross-sectional moment and polar moment are considered to be bone parameters that will provide valuable information to elucidate bone quality.

## Conclusions

We have demonstrated that single fasting for 96 h causes a difficult-restoring bone quality change in the rat lumbar vertebrae.

## Supplemental Information

10.7717/peerj.6161/supp-1 Supplemental Information 1 Raw data corresponding to each figure.

## Additional Information and Declarations

### Data Availability

The following information was supplied regarding data availability: The original imaging data obtained from the X-ray CT system (Latheta LCT-100; Hitachi Aloka Medical) is available at Figshare: Hisatomi, Yuko; Kugino, Kenji (2018): Changes in bone density and bone quality caused by single short-term severe fasting in rats. figshare. Dataset. DOI 10.6084/m9.figshare.7159523.v1.
